# Influence of the 2-methylimidazole/zinc nitrate hexahydrate molar ratio on the synthesis of zeolitic imidazolate framework-8 crystals at room temperature

**DOI:** 10.1038/s41598-018-28015-7

**Published:** 2018-06-25

**Authors:** Yongyong Zhang, Ying Jia, Ming Li, Li’an Hou

**Affiliations:** Xi’an High Technology Institute, Xi’an, China

## Abstract

The effect of the 2-methylimidazole (Hmim)/zinc nitrate hexahydrate (Zn) molar ratio on the physicochemical characteristics of the zeolitic imidazolate framework-8 (ZIF-8) was investigated. ZIF-8 crystals were synthesized by mixing Hmim with Zn at room temperature without any additives in methanol solution. It was found that Hmim/Zn molar ratio had significant influence on the crystallinity, yield, particle size and porosity of ZIF-8. The samples synthesized at low Hmim/Zn molar ratio showed a cubic shape, whereas at higher Hmim/Zn ratios truncated rhombic dodecahedron or rhombic dodecahedron morphologies were obtained. The particle size is decreased upon increasing the Hmim/Zn molar ratio. Besides, higher Hmim/Zn molar ratio in a certain range resulted in improving crystallinity, yield, surface area and micropore volume of ZIF-8. The ZIF-8 crystals produced at Hmim/Zn molar ratio of 8 exhibited the best characteristics. The present work provides new insights in relation to the role of Hmim/Zn molar ratio on the synthesis process of ZIF-8.

## Introduction

As a subclass of metal organic frameworks (MOFs), zeolitic imidazolate frameworks (ZIFs) are porous crystals with zeolite-type structure built by metal ions and imidazolate ligands^1^. ZIFs possess the unique properties of high crystallinity, large surface area, exceptional chemical and functional tunability. As a result, ZIFs have gained considerable attention for their potential application in gas storage^2^, CO_2_ capture^3,4^, separation^5,6^, sensing^7^, catalysis^8^, drug delivery^9,10^ and water treatment^11,12^. Zeolitic imidazolate framework-8 (ZIF-8), with sodalite (SOD) topology constructed from Zn and Hmim, is one of the most investigated ZIF materials^13–15^. Nowadays, ZIF-8 can be synthesized using a variety of methods including solvothermal method^1^, aqueous synthesis at room temperature^16^, microwave-assisted method^17,18^, vapor-assisted conversion method^19^, ultrasonic synthesis^20^, electrochemical routines^21^, mechanochemical synthesis^22^ and reverse microemulsion method^23^. Aqueous synthesis at room temperature has been widely employed owing to its simplicity, short times and energy saving. The properties of ZIF-8 are related to many factors such as reaction time, solvent, zinc salts, molar ratio and additives. Zhu *et al*.^24^ and Venna *et al*.^25^ investigated the influence of reaction time on the crystal growth without any additives in methanol solution. Eugenia *et al*.^26^ explored the effect of the solvent on the synthesis process and on the nanocrystal characteristics of ZIF-8. Schejn *et al*.^27^ reported on the impact of the zinc source on the morphology and size of ZIF-8 in methanol at room temperature. Jian *et al*.^28^ systematically studied the influence of zinc sources, concentration of Hmim, Hmim/Zn molar ratio and water content on the morphology, particle size, and crystallinity of ZIF-8 at room temperature in water solution. However, few studies have been carried out to systematically explore the influence of Hmim/Zn molar ratio on the properties of ZIF-8 without any additives in methanol solution at room temperature.

Herein, ZIF-8 crystals were synthesized by mixing 2-methylimidazole (Hmim) with zinc nitrate hexahydrate (Zn) at room temperature without any additives in methanol solution. The effect of the Hmim/Zn molar ratio on the properties of ZIF-8 was investigated. A complimentary characterization study, involving X-ray diffraction (XRD), scanning electron microscopy (SEM), energy dispersive spectroscopy (EDS), Fourier transform infrared spectroscopy (FTIR) and N_2_ adsoprtion-desorption was carried out to investigate the physicochemical characteristics of ZIF-8 samples.

## Results and Discussion

### XRD analysis

**Figure 1:**
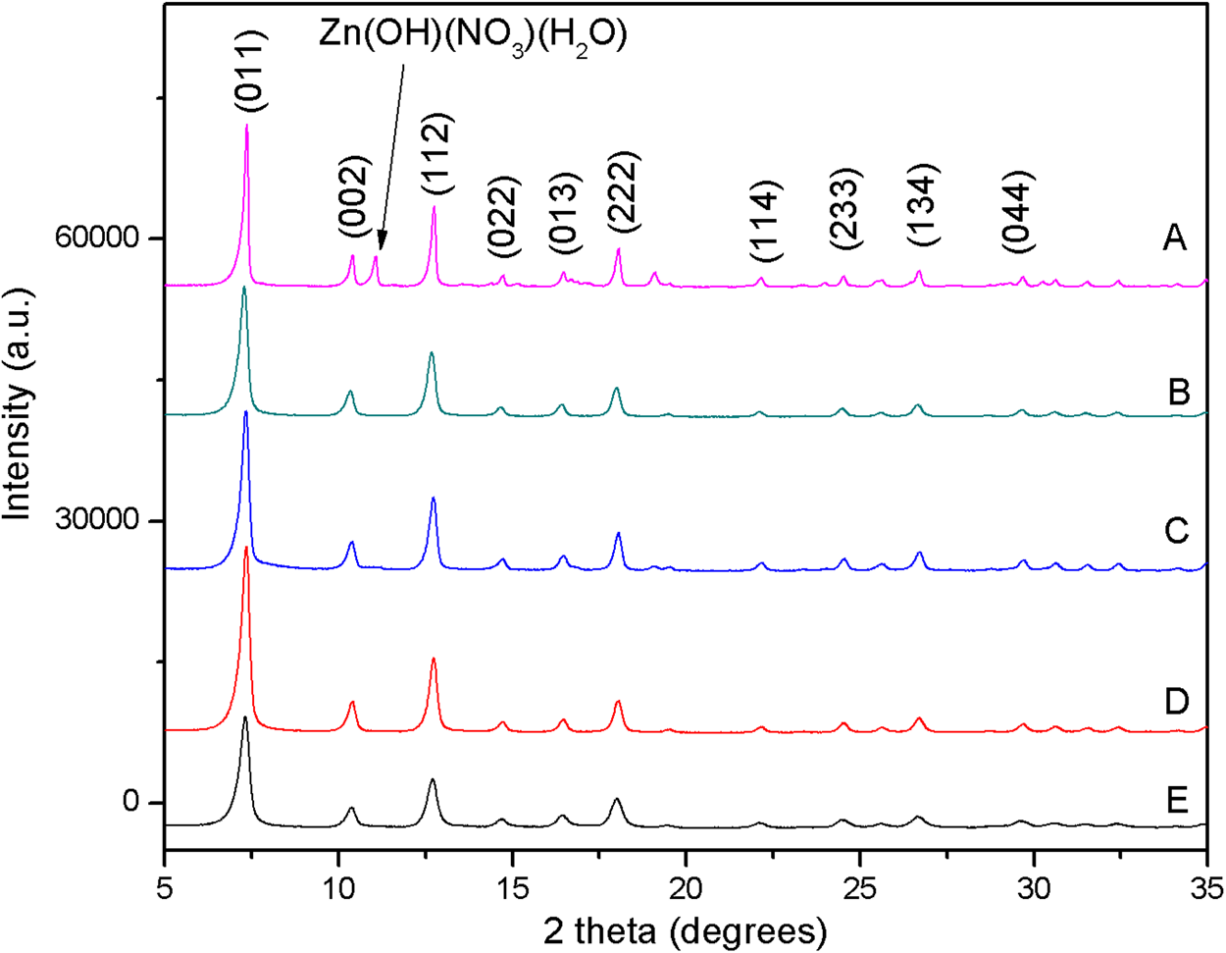
XRD patterns of the different ZIF-8 samples prepared at Hmim/Zn molar ratio of (**A**) 1, (**B**) 2, (**C**) 4, (**D**) 8 and (**E**) 16.

Figure 1 depicts the XRD spectra of the different ZIF-8 samples prepared in the present work by altering the Hmin/Zn ratio (Table 1). The characteristic diffraction peaks of ZIF-8 at 2θ = 7.4°, 10.4°, 12.7°, 14.7°, 16.4°, 18.0°, 22.1°, 24.5°, 26.7° and 29.6°, which corresponded to planes of (011), (002), (112), (022), (013), (222), (114), (233), (134) and (044) respectively, can be clearly seen. The prominent reflections agreed well with previous reports, confirming the typical sodalite structure of ZIF-8^1,27,29^. Clearly, some diffraction peaks of Zn(OH)(NO_3_)(H_2_O) and other unkown phases were observed in the XRD patterns of ZIF-8 synthesized at the Hmim/Zn molar ratio of 1. This result was consistent with observations of Kida *et al*.^16^ and Chen *et al*.^30^. The intensity of diffraction peaks increased with the increasing amount of Hmim, reaching maximum at Hmim/Zn molar ratio of 8. The latter indicates that high Hmim/Zn molar ratio are required in order to obtained high crystallinity of ZIF-8.

**Table 1:** Relative crystallinity and yield of ZIF-8 prepared at different Hmim/Zn molar ratio.

Samples	Hmim/Zn molar ratio	Relative crystallinity (%)	Yield (%)
A	1	73.13	25.65
B	2	74.56	32.11
C	4	92.07	40.82
D	8	100	51.09
E	16	75.68	49.46

As shown in Table 1, the relative crystallinity and yield of ZIF-8 gradually increased as the Hmim/Zn molar ratio increased from 1 to 8. For higher Hmin/Zn molar ratios, i.e. 16, a decrease in the relative crystallinity and yield was observed, implying an optimum Hmim/Zn molar ratio equal to 8. This can be attributed to the coverage of particle’s surface by excess Hmim, which hinders particle growth. Similar results have been obtained by Daigo *et al*.^31^.

### SEM/EDS analysis

**Figure 2:**
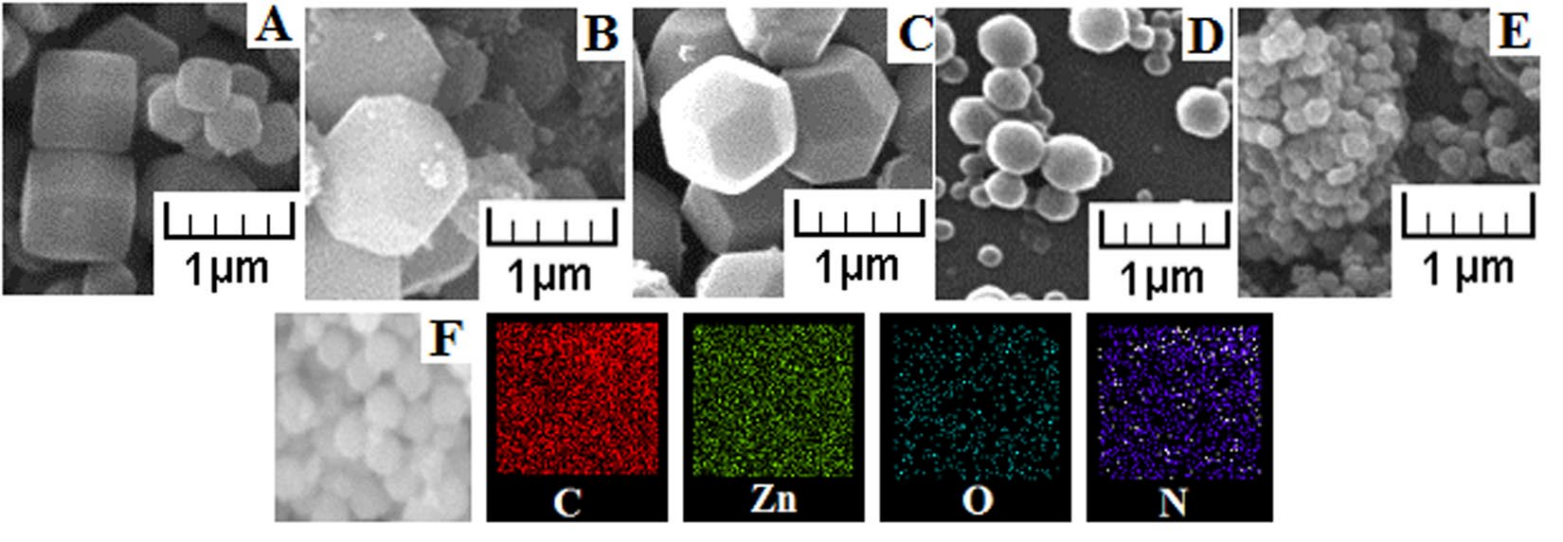
SEM images of the different ZIF-8 samples prepared at Hmim/Zn molar ratio of (**A**) 1, (**B**) 2, (**C**) 4, (**D**) 8, (**E**) 16 and elemental mapping of ZIF-8 with Hmim/Zn molar ratio of 8.

Figure 2 displays the morphology of ZIF-8 at different Hmim/Zn molar ratios and the corresponding elemental mapping. ZIF-8 synthesized at Hmim/Zn molar ratio of 1 had a cubic shape, with particles size of 0.4 μm and 0.8 μm. For Hmim/Zn molar ratios higher than 1, all samples exhibit truncated rhombic dodecahedron (B and C) or rhombic dodecahedron (D and E) morphologies. The average particle size gradually decreased from 1 μm to 0.1 μm as Hmim/Zn molar ratio increased from 4 to 16, respectively. A similar trend in relation to the change of ZIF-8 size as a function of Hmim/Zn molar ratio, has been observed in other reports^16,28,32^. Moreover, an excess of Hmim results in particles aggregation as shown in Fig. 2(E). Elemental mapping demonstrated homogeneously distribution of C, Zn and N elements in ZIF-8. The O element may come from the residue of zinc nitrate hexahydrate or the conductive adhesive used in the characterization.

### Textural analysis

**Figure 3:**
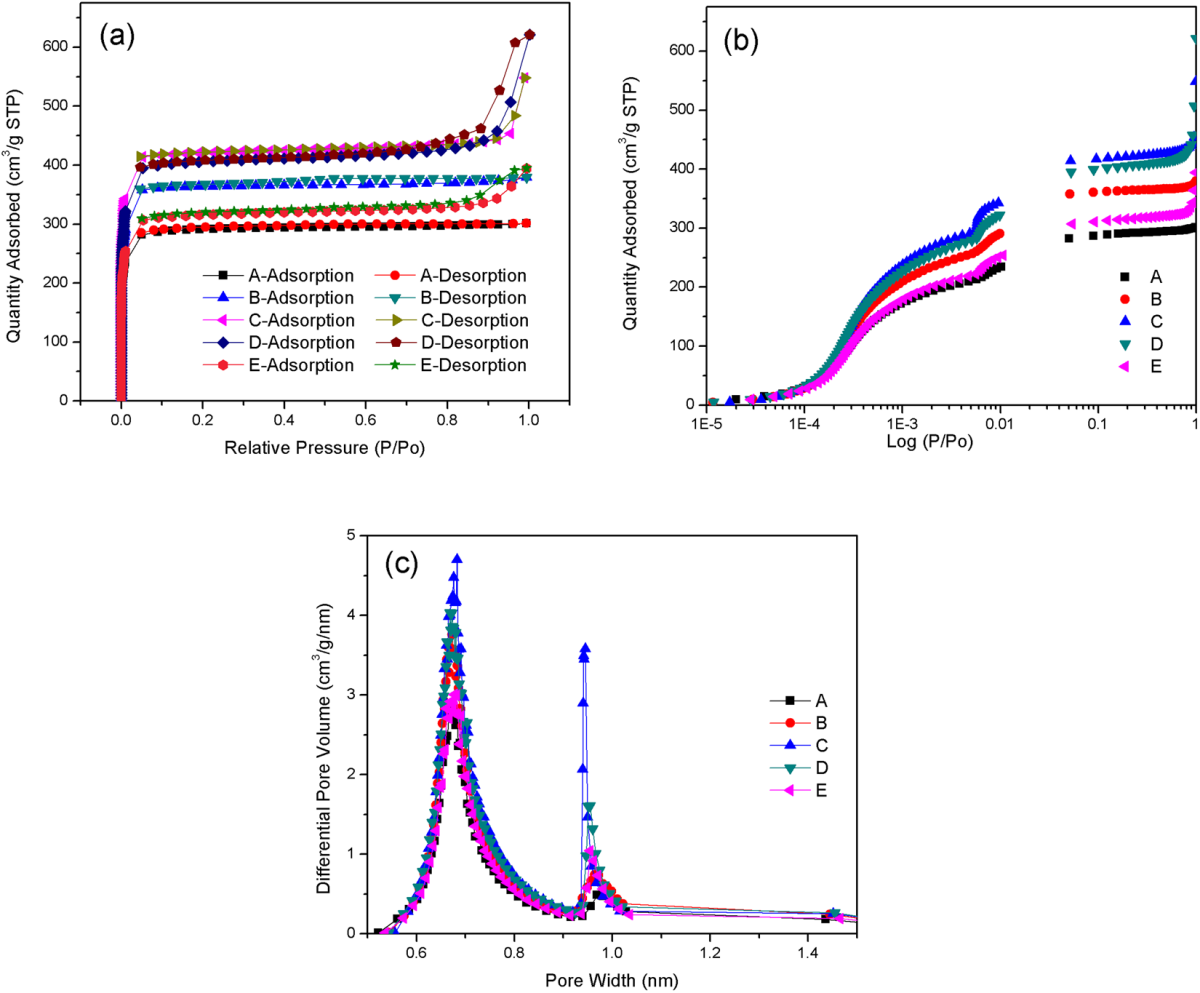
(**a**) Nitrogen adsorption-desorption isotherms of the different ZIF-8 samples prepared at Hmim/Zn molar ratio of (A) 1, (B) 2, (C) 4, (D) 8 and (E) 16, (**b**) logarithmic-scale plot of nitrogen adsorption and (**c**) corresponding pore size distributions.

As shown in Fig. 3(a), the nitrogen adsorption-desorption isotherms of ZIF-8 samples prepared at different Hmim/Zn molar ratio display typical reversible type I isotherms. The adsorbed nitrogen in all samples at low pressures (*P*/*P*_0_ < 0.08) increased steeply, suggesting the existence of micropores. The results were in line with those reported previously^29,33,34^. Furthermore, the isotherms of ZIF-8 prepared at Hmim/Zn molar ratio of 4 and 8 exhibited hysteresis loop near *P*/*P*_0_ = 1, indicating the presence of interparticle mesoporosity and macroporosity between ZIF-8 particles^35^, in consistent with SEM results. Indeed, the semi-log plot of nitrogen adsorption isotherms of ZIF-8 showed two steps occurring at 5 × 10^−2^ and 5 × 10^−3^ *P*/*P*_0_ (Fig. 3(b)). The first step could be attributed to a reorganization of the nitrogen molecules caused by strong electrostatic interactions with the ZIF framework, while the second step to the gas-induced rotation of the imidazolate linkers upon pressure, i.e. to the gate-opening effect^36^. The increase in uptake going from the first to the second step was 24.8%, 27.4%, 28.6%, 28.7% and 27.1% at Hmim/Zn molar ratio of 1, 2, 4, 8 and 16, respectively. This indicated that Hmim/Zn molar ratio had no significant influence on the gate-opening effect of ZIF-8.

The pore size distribution (PSD) determined by the Horvath–Kawazoe method were presented in Fig. 3(c). All the samples displayed the same PSD curves with two main peaks at 0.67 nm and 0.96 nm. Interestingly, the sample with Hmim/Zn molar ratio of 8 possesses the highest micropores at a pore width of 0.67 nm and 0.96 nm, in good agreement with the t-plot micropore volume (Table 2).

**Table 2:** Porosity properties of ZIF-8 prepared in this study and other references.

Samples	Hmim/Zn molar ratio	Uptake of N_2_ (i) (cm^3^/g STP)	Uptake of N_2_ (ii) (cm^3^/g STP)	BET surface area (m^2^/g)	Langmuir surface area (m^2^/g)	Micropore volume^a^ (cm^3^/g)
A	1	212	282	880	1295	0.42
B	2	260	358	1097	1613	0.54
C	4	282	395	1228	1897	0.59
D	8	295	414	1268	1943	0.62
E	16	223	306	955	1449	0.45
Reference						
^11^	10.8	—	—	1237	—	0.66
^27^	8	—	—	1700 ± 30	—	0.66
^33^	7.9	—	—	1386	—	0.61
^37^	8	—	—	1258	—	0.62

^a^T-plot method.

Table 2 summurizes the Brunauer-Emmett-Teller (BET) parameters, Langmuir surface area and the micropore volume calculated with t-plot method. The maximum BET surface area, Langmuir surface area and micropore volume of 1268 m^2^/g, 1943 m^2^/g and 0.62 cm^3^/g, respectively were obtained for the sample synthesized at Hmim/Zn molar ratio of 8. These results are in agreement to the values reported by Dai *et al*.^11^ and Wu *et al*.^37^, but lower than these reported by Schejn *et al*.^27^ and Li *et al*.^33^. This is mainly due to the different synthesis conditions as well as to the different pre-treatment before N_2_ adsorption. In the present work, the pre-treatment processes (such as the washing step^38^ and the evacuation conditions) may lead to the relatively lower values.

Upon increasing the molar ratio of Hmim/Zn, the BET, Langmuir surface area and micropore volume increased, reaching maximum at Hmim/Zn molar ratio of 8. Then the surface area and micropore volume decreased gradually with the increase of Hmim/Zn molar ratio. These results can be interpreted taking into account that low Hmim/Zn molar ratios facilitates the formation of by-products possessing small surface areas^16^, whereas at high Hmin/Zn ratios the Hmin excess could lead to the decrease of surface area and micropore volume^29^.

### FTIR spectroscopy analysis

**Figure 4:**
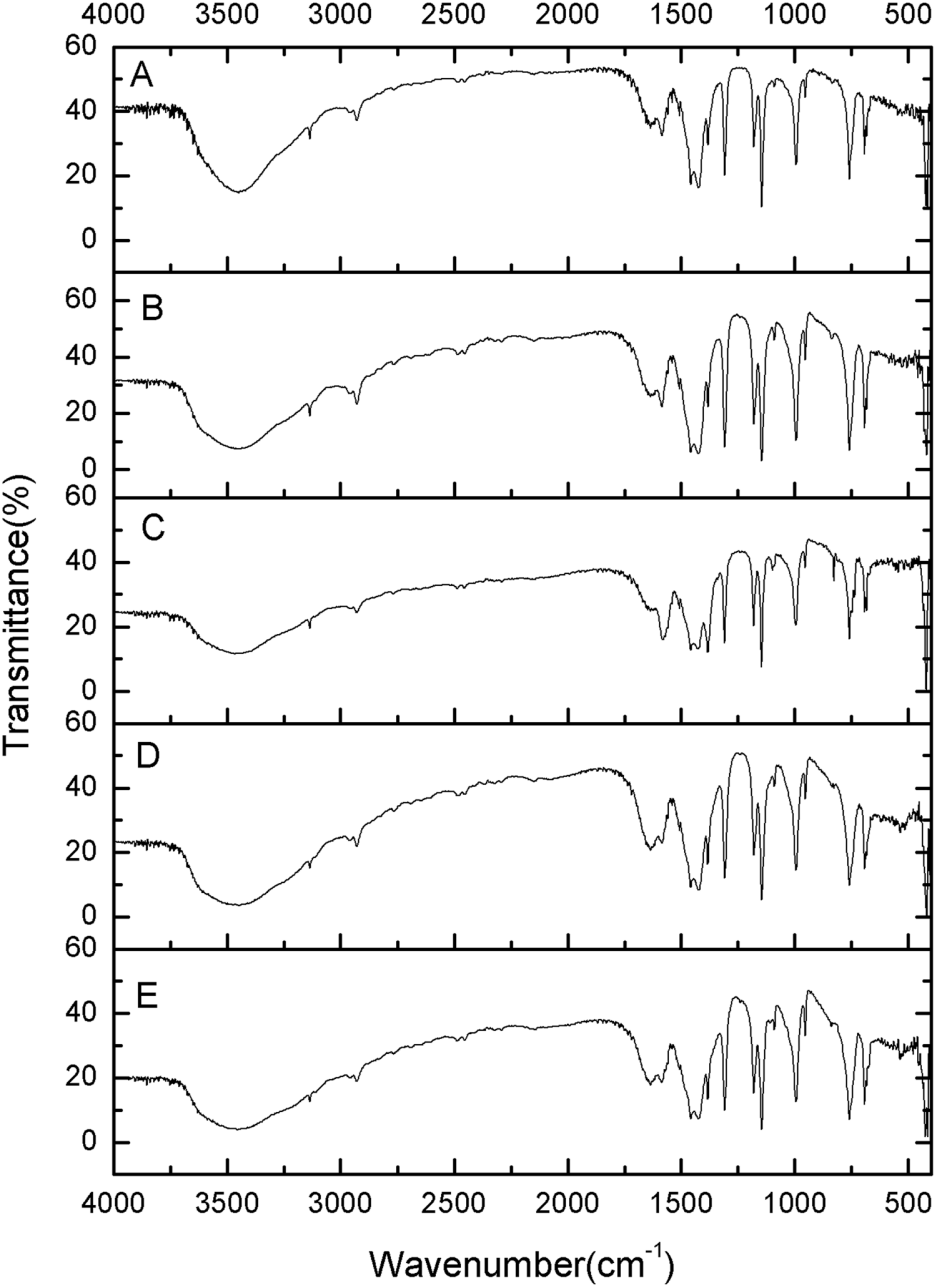
FT-IR spectra of the different ZIF-8 samples prepared at Hmim/Zn molar ratio of (A) 1, (B) 2, (C) 4, (D) 8 and (E) 16.

The FT-IR spectra of ZIF-8 samples prepared at different Hmim/Zn molar ratios are shown in Fig. 4. The main bands at 3455, 3135, 2929, 1635, 1585, 1458, 1425, 1385,1309, 1146, 995, 760, 694 and 426 cm^−1^ can be seen in all samples. These bands are in consistent with those previously reported by Cravillon *et al*.^39^, Ordonez *et al*.^40^ and Jomekian *et al*.^41^. The band at 3455 cm^−1^ might be attributed to the N–H stretching vibration of the residual Hmim and the O–H stretching vibration of water from KBr deliquescence. The peaks at 3135 and 2929 cm^−1^ were associated with the aromatic and aliphatic C–H asymmetric stretching vibrations, respectively. The band around 1635 cm^−1^ arose from the C=C stretch mode, while band at 1585 cm^−1^ corresponded to the C=N stretch vibration. The signals at 1300–1460 cm^−1^ were for the entire ring stretching, whereas the band at 1146 cm^−1^ derived from aromatic C–N stretching mode. Similarly, the peaks at 995 and 760 cm^−1^ could be assigned to C–N bending vibration and C–H bending mode, respectively. The band at 694 cm^−1^ was due to the ring out-of-plane bending vibration of the Hmim. Interestingly, the Zn–N stretching vibration band at 426 cm^−1^ was clearly observed, demonstrating the chemical combination of zinc ions with nitrogen atoms of the methylimidazole groups towards the formation of imidazolate^42^. No significant differences on the chemical functional groups of ZIF-8 samples prepared at different Hmim/Zn molar ratio were recorded, signifying the minor role of Hmim/Zn molar ratio on the building units of ZIF-8.

In conclusion, phase-pure ZIF-8 particles were successfully synthesized from zinc nitrate hexahydrate and 2-methylimidazole in methanol at room temperature without using any additives. The relative crystallinity, yield, particle size and porosity properties were markedly affected by the Hmim/Zn molar ratio in the same reaction time. Zinc hydroxide was detected only at low Hmim/Zn molar ratios, as demonstrated by XRD measurements. The particle’s size in considerably decreased as the Hmim/Zn molar ratio increased. Moreover, higher Hmim/Zn molar ratio resulted in improved crystallinity, yield, surface area and t-plot micropore volume of ZIF-8. The ZIF-8 crystals produced at Hmim/Zn molar ratio of 8 exhibited the highest crystallinity, yield (51.09%), surface area (BET: 1268 m^2^/g, Langmuir: 1943 m^2^/g) and t-plot micropore volume (0.62 cm^3^/g). At higher than 8 molar ratios, the Hmim excess inhibited particle growth, bringing out the decrease of crystallinity, surface area, porosity and yield. The present results demonstrate the importance of the Hmim/Zn molar ratio in the crystallization kinetics and evolution of ZIF-8 crystals.

## Experimental Methods

### Materials

Zinc nitrate hexahydrate (Zn(NO_3_)_2_·6H_2_O, >99.0%) and 2-methylimidazole (Hmim, >98.0%) were purchased from Sigma Aldrich. Methanol (MeOH, >99.5%) was obtained from Guangzhou Jinhuada chemical reagent Co., Ltd. All chemicals were of analytical grade and used without any purification. Deionized water was used throughout this work.

### Preparation of ZIF-8

Typically, 1.484 g of Zn(NO_3_)_2_·6H_2_O was first dissolved in 50 mL MeOH, which was labeled as solution A. 3.278 g Hmim was dissolved in 50 mL MeOH, which was labeled as solution B. To examine the effects of the Hmim/Zn molar ratio on the synthesized products, the Hmim/Zn molar ratio was adjusted from 1 to 16 by adjusting the concentration of Hmim at a fixed Zn concentration. The solution B was slowly poured into solution A under stirring at room temperature (25 ± 3 °C). The mixture solution was stirred for 60 min at constant speed of 200 rpm. Then the product was collected from the colloidal dispersion by centrifugation (4000 rpm, 30 min) and washed with MeOH three times. Finally, the obtained products were dried in air for subsequent characterization.

### Characterization

X-ray diffraction (XRD, X’PERT MRD, PANalytic, Holland) was performed using Cu Kα radiation at 40 kV and 50 mA to determine the crystalline. The 2θ scanning range was 5–35°, the step size was 0.008°(2θ) and the scanning speed was 3°(2θ)/min. The relative crystallinity was determined based on the integrated peak areas of samples at 2θ = 7.4°, 10.4°, 12.7°, 14.7°, 16.4° and 18.0°, and considering 100% crystallinity for the one with the strongest peaks (ZIF-8 prepared at Hmim/Zn molar ratio of 8). The peak areas were quantified using the MDJ Jade software after baseline correction. And the yield was calculated based on the amount of zinc. Scanning electron microscopy (SEM, TESCAN VEGAІІ XMUINCN, Czech Republic) in combination with energy dispersive spectrometer (EDS, OXFORD INCA) were used to characterize the morphology and particles size. The accelerating voltage and current of electron beam are 21 keV and 0.2 nA respectively. Each sample was coated with a very thin gold using a sputter coater under certain high-vacuum conditions to achieve conductivity and vacuum durability and then put into the SEM chamber. The surface area and adsorption–desorption isotherm measurements were carried out on a Quantachrome Autosorb-iQ gas sorption instrument at 77 K using liquid nitrogen as coolant. The sample was degassed at 150 °C for 3 h under vacuum before the measurements. The textural properties were determined via nitrogen sorption at −196 °C. Fourier transform infrared (FTIR) spectra in the range 4000−400 cm^−1^ were obtained on a Thermo Fisher Nicolet 6700 FTIR spectrometer using the KBr wafer technique.

## References

[1] Park KS (2006). Exceptional chemical and thermal stability of zeolitic imidazolate frameworks. Proceedings of the National Academy of Sciences of the United States of America.

[2] Villajos JA, Orcajo G, Calleja G, Botas JA, Martos C (2016). Beneficial cooperative effect between Pd nanoparticles and ZIF-8 material for hydrogen storage. International Journal of Hydrogen Energy.

[3] Nguyen NTT (2016). Mixed-metal zeolitic imidazolate frameworks and their selective capture of wet carbon dioxide over methane. Inorganic Chemistry.

[4] Marti AM (2017). Continuous flow processing of ZIF-8 membranes on polymeric porous hollow fiber supports for CO_2_ capture. ACS Applied Materials & Interfaces.

[5] Cacho-Bailo F, Seoane B, Teĺlez C, Coronas J (2014). ZIF-8 continuous membrane on porous polysulfone for hydrogen separation. Journal of Membrane Science.

[6] Abolfazl J, Bahamin B, Reza MB, Toraj M, Ali K (2017). Ionic liquid-modified Pebax® 1657 membrane filled by ZIF-8 particles for separation of CO2 from CH4, N2 and H2. Journal of Membrane Science.

[7] Gassensmith JJ (2014). A metal-organic framework-based material for electrochemical sensing of carbon dioxide. Journal of the American Chemical Society.

[8] Thanh T, Tam MH, Chung KN, Quynh TNH, Nam TSP (2015). Expanding applications of zeolite imidazolate frameworks in catalysis: synthesis of quinazolines using ZIF-67 as an efficient heterogeneous catalyst. RSC Advances.

[9] Tian ZF, Yao XX, Zhu YF (2017). Simple synthesis of multifunctional zeolitic imidazolate frameworks-8/graphene oxide nanocrystals with controlled drug release and photothermal effect. Microporous and Mesoporous Materials.

[10] Harpreet K, Girish CM, Vandana G, Deepak K, Sachin T (2017). Synthesis and characterization of ZIF-8 nanoparticles for controlled release of 6-mercaptopurine drug. Journal of Drug Delivery Science and Technology.

[11] Dai J (2018). Synthesis of novel microporous nanocomposites of ZIF-8 on multiwalled carbon nanotubes for adsorptive removing benzoic acid from water. Chemical Engineering Journal.

[12] Zahra A (2016). Effect of carbonization temperature on adsorption property of ZIF-8 derived nanoporous carbon for water treatment. Microporous and Mesoporous Materials.

[13] Trung TB, Nguyen DC, Yong SK, Hyungphil C (2018). *In situ* growth of microporous ZIF-8 nanocrystals on a macroporous phyllosilicate mineral. Materials Letters.

[14] Satoshi W (2017). Synthesis of zeolitic imidazolate framework-8 particles of controlled sizes, shapes, and gate adsorption characteristics using a central collision-type microreactor. Chemical Engineering Journal.

[15] Oleksii K (2017). Microfluidic reactors for the size-controlled synthesis of ZIF-8 crystals in aqueous phase. Materials and Design.

[16] Koji K, Muneyuki O, Kosuke F, Shunsuke T, Yoshikazu M (2013). Formation of high crystalline ZIF-8 in an aqueous solution. CrystEngComm.

[17] Bao QL, Lou YB, Xing TT, Chen JX (2013). Rapid synthesis of zeolitic imidazolate framework-8 (ZIF-8) in aqueous solution via microwave irradiation. Inorganic Chemistry Communications.

[18] Jeong HK (2017). Rapid microwave-assisted synthesis of hybrid zeolitic-imidazolate frameworks (ZIFs) with mixed metals and mixed linkers. Journal of Materials Chemistry A.

[19] Zhang H, Shi Q, Kang XZ, Dong JX (2013). Vapor-assisted conversion synthesis of prototypical zeolitic imidazolate framework-8. Journal of Coordination Chemistry.

[20] Cho HY, Kim J, Kim SN, Ahn WS (2013). High yield 1-L scale synthesis of ZIF-8 via a sonochcmical route. Microporous and Mesoporous Materials.

[21] Joaristi AM, Juan-Alcaniz J, Serra-Crespo P, Kapteijn F, Gascon J (2012). Electrochemical synthesis of some archetypical Zn^2+^, Cu^2+^, and Al^3+^ metal organic frameworks. Crystal Growth & Design.

[22] Beldon PJ (2010). Rapid room temperature synthesis of zeolitic imidazolate frameworks by using mechanochemistry. Angewandte Chemie-International Edition.

[23] Sun WZ, Zhai XS, Zhao L (2016). Synthesis of ZIF-8 and ZIF-67 nanocrystals with well-controllable size distribution through reverse microemulsions. Chemical Engineering Journal.

[24] Zhu MQ, Venna SR, Jasinski JB, Carreon MA (2011). Room-temperature synthesis of ZIF-8: the coexistence of ZnO nanoneedles. Chemistry of Materials.

[25] Venna SR, Jasinski JB, Carreon MA (2010). Structural evolution of zeolitic imidazolate framework-8. Journal of the American Chemical Society.

[26] Eugenia LB, José LF, Juan MZ (2014). Influence of the solvent in the synthesis of zeolitic imidazolate framework-8 (ZIF-8) nanocrystals at room temperature. Journal of Colloid and Interface Science.

[27] Schejn A (2014). Controlling ZIF-8 nano- and microcrystal formation and reactivity through zinc salt variations. CrystEngComm.

[28] Jian MP (2015). Water-based synthesis of zeolitic imidazolate framework-8 with high morphology level at room temperature. RSC Advances.

[29] Pan YC, Liu YY, Zeng GF, Zhao L, La ZP (2011). Rapid synthesis of zeolitic imidazolate framework-8 nanocrystals in an aqueous system. Chemical Communications.

[30] Chen BL, Bai FH, Zhu YQ, Xia YD (2014). A cost-effective method for the synthesis of zeolitic imidazolate framework-8 materials from stoichiometric precursors via aqueous ammonia modulation at room temperature. Microporous and Mesoporous Materials.

[31] Daigo Y (2013). Synthesis and adsorption properties of ZIF-8 nanoparticles using a micromixer. Chemical Engineering Journal.

[32] Shunsuke T, Koji K, Muneyuki O, Yosuke I, Yoshikazu M (2012). Size-controlled synthesis of zeolitic imidazolate framework-8 (ZIF-8) crystals in an aqueous system at room temperature. Chemistry Letters.

[33] Li SL, Yin FY, Noraishah CA, Kok KL, Azmi MS (2014). Effect of synthesis parameters on the formation of Zeolitic Imidazolate Framework 8 (ZIF-8) nanoparticles for CO_2_ adsorption. Particulate Science and Technology.

[34] He M (2014). Facile synthesis of zeolitic imidazolate framework-8 from a concentrated aqueous solution. Microporous and Mesoporous Materials.

[35] Liu D, Wu Y, Xia Q, Li Z, Xi. H (2013). Experimental and molecular simulation studies of CO_2_ adsorption on zeolitic imidazolate frameworks: ZIF-8 and amine-modified ZIF-8. Adsorption.

[36] Fairen-Jimenez D (2011). Opening the gate: framework flexibility in ZIF-8 explored by experiments and simulations. Journal of the American Chemical Society.

[37] Wu YN (2014). Amino acid assisted templating synthesis of hierarchical zeolitic imidazolate framework-8 for efficient arsenate removal. Nanoscale.

[38] Demessence A (2010). Adsorption properties in high optical quality nanoZIF-8 thin films with tunable thickness. Journal of Materials Chemistry.

[39] Cravillon J (2009). Rapid room-temperature synthesis and characterization of nanocrystals of a prototypical zeolitic imidazolate framework. Chemistry of Materials.

[40] Ordonez MJC, Balkus KJ, Ferraris JP, Musselman IH (2010). Molecular sieving realized with ZIF-8/Matrimid (R) mixed-matrix membranes. Journal of Membrane Science.

[41] Jomekian A, Behbahani RM, Mohammadi T, Kargari A (2016). Innovative layer by layer and continuous growth methods for synthesis of ZIF-8 membrane on porous polymeric support using poly(ether-block-amide) as structure directing agent for gas separation. Microporous and Mesoporous Materials.

[42] Hu Y, Kazemian H, Rohani S, Huang Y, Song Y (2011). *In situ* high pressure study of ZIF-8 by FTIR spectroscopy. Chemical Communications.

